# Elimination of Hole Mouth Burr in Multilayer PCB Micro-Hole by Using Micro-EDM

**DOI:** 10.3390/mi12060688

**Published:** 2021-06-12

**Authors:** Xinke Feng, Bin Xu, Jianguo Lei, Xiaoyu Wu, Feng Luo, Lianyu Fu

**Affiliations:** 1 Shenzhen Key Laboratory of High Performance Nontraditional Manufacturing, Shenzhen University, Shenzhen 518060, China; fengxinke2019@email.szu.edu.cn (X.F.); leijg@szu.edu.cn (J.L.); wuxy@szu.edu.cn (X.W.); LLF@szu.edu.cn (F.L.); 2 Shenzhen Jinzhou Precision Technology Corp., Shenzhen 518116, China; mlyfu@chinadrill.com

**Keywords:** multilayer PCB, micro-hole, hole mouth burr, micro-EDM

## Abstract

The micro-hole is a key structure in multilayer printed circuit board (PCB), as it enables the effective transmission of electrical signals. At present, the most common way to machine PCB micro-holes is mechanical drilling using micro-bit. However, in the mechanical drilling of micro-holes, these holes are prone to burring at the hole mouth due to the micro-bit failing to cleanly cut through the first layer of copper foil on the PCB. Hole mouth burr can seriously affect the performance of the PCB, resulting in potential short circuiting or even ruining the PCB. To solve the above problems, this paper proposed to machine the first layer of copper foil on the PCB via micro electro-discharge machining (micro-EDM) to eliminate hole mouth burr. Compared with the mechanical drilling, micro-EDM is a form of non-contact machining, and the high temperature generated from the electric spark discharge can erode the first layer of copper foil, thus fully eliminating hole mouth burr. This paper performed a detailed study of the influence of spindle speed, machining voltage, pulse width, and pulse interval on hole mouth quality. After that, the technological parameters for eliminating hole mouth burr were obtained. Finally, under the effects of 20,000 rpm spindle speed, 26 V machining voltage, 4 μs pulse width, and 8 μs pulse interval, a micro-bit with a diameter of 200 μm was used to perform micro-EDM of the first layer of copper foil. From the machining results, it can be known that the PCB micro-hole was possessed of overall good quality, with good hole wall surface quality and almost no visible hole mouth burr.

## 1. Introduction

Due to spatial restrictions in product design, multilayer printed circuit board (PCB) has been widely used in electronic products [1]. In multilayer PCB, micro-holes can be used to enable the transmission of electrical signals, so they are the important structure of multilayer PCB. At present, the main way to machine PCB micro-holes is mechanical drilling by using micro-bits. However, during the mechanical drilling, PCB micro-holes are prone to burring around the edge of the hole due to the micro-bit failing to cleanly cut through the first layer of copper foil on the PCB [2]. The hole mouth burr can seriously affect PCB performance, potentially resulting in short circuiting or even ruining the PCB [3]. Researchers from all over the world have carried out extensive research on the mechanical drilling of PCB micro-holes from the perspectives of chip removal [4] and drill wear [5] through simulation analysis [6] or experiments [7]. Ranjan et al. proposed an artificial-intelligence-based method to predict hole quality and tool breakage through comprehensive analysis of thrust force, torque, and vibration signals [8]. Previous studies have shown that, when mechanical drilling of PCB micro-holes was carried out, increasing the spindle speed and reducing the feed speed can reduce the size of hole mouth burr [9], but cannot completely eliminate it. There is another PCB drilling technology, laser drilling, which is widely used in the processing of micro-holes [10]. However, the walls of the micro-holes processed by laser drilling are usually tapered [11], and the problem of poor quality of the hole mouth has yet to be solved. Compared with the mechanical drilling, micro electro-discharge machining (micro-EDM) is a form of non-contact machining, and the high temperature generated from the electric spark discharge in this process can erode the first layer of copper foil, thus eliminating the hole mouth burr.

Micro-EDM is a non-contact machining process [12] that generates high temperatures through high-frequency discharges between the tool electrode and workpiece and erodes the workpiece material [13]. Due to micro-EDM’s advantages of burr-free [14], small cutting force and the ability to machine very hard materials [15] and extremely small holes [16], it has been widely used in the machining of micro-holes [17]. D’Urso et al. [18] carried out a machining experiment of micro-holes on stainless steel plates with micro-EDM. Based on the experimental results, they found that increasing the conductivity of the electrode and the voltage was able to increase the material removal rate. In order to prevent back-strike in the breakout stage when EDM was used to drill workpieces with inner channels or cavities, Zhang et al. [19] proposed a new control strategy to detect the hole completion, which was able to accurately identify the completion status of the hole and improve drilling efficiency. For promoting the circulation of dielectric and removal of debris in micro-EDM of micro-holes, Anthuvan et al. [20] applied magnetic fields in the sparking region and added activated carbon powder to the dielectric medium. Their experimental result showed that both the material removal rate and tool wear rate were increased in that hybrid process. When machining micro-holes with high aspect ratio in micro-EDM, the assistance of ultrasonic vibration was also considered to promote the removal of debris, which was supported by Liu et al. [21] through simulation analysis. Ni et al. [22] machined small, deep holes on Inconel 718 and 41Cr4 workpieces by combining ultrasonic vibration and electrode rotation and forced pump flushing, which could increase machining stability and improve the material removal rate.

**Figure 1:**
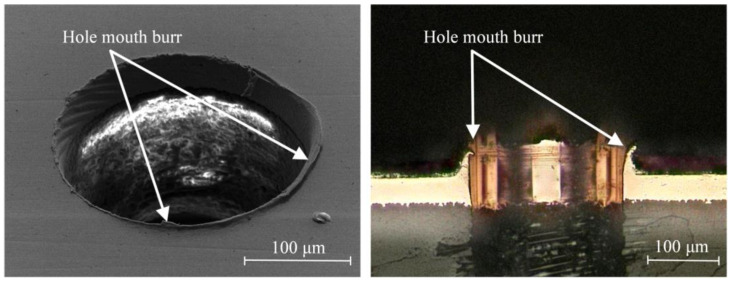
Hole mouth burr in printed circuit board (PCB) micro-hole.

Based on the above-mentioned comparative analysis of the characteristics of traditional PCB micro-hole machining technology and micro-EDM technology, the purpose of this paper was to eliminate PCB micro-hole burrs (see Figure 1) by using micro-EDM to machine the first layer copper foil of PCB. This research might provide new possibilities for the development of PCB micro-hole machining technology.

## 2. Technological Process

**Figure 2:**
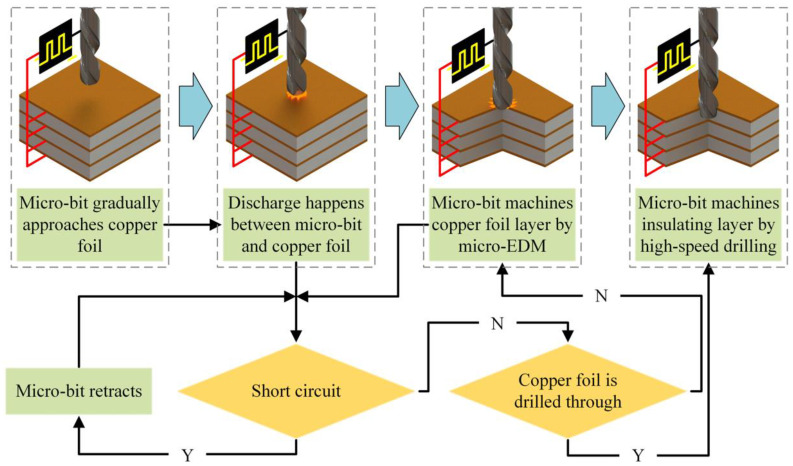
Specific technical process for machining PCB micro-hole by using micro electro-discharge machining (micro-EDM).

A multilayer PCB is composed of copper foil, modified resin, ceramic packing, and glass fiber cloth, which are superimposed layer by layer (see Figure 2). In mechanical drilling of the first layer of copper foil, the first layer of copper foil will be cut to form chips under the action of shear force. However, when the diameter of the hole is very small, under the effects of uneven copper foil material and nonstandard cutting-edge shapes, the copper foil is unable to be neatly cut. In addition, under the extrusive force of the micro-bit, chips that are not cut cleanly will undergo plastic deformation and adhere to the hole mouth, thus forming the hole mouth burr [23].

Compared with the mechanical drilling process, the EDM process can avoid the plastic deformation of the copper foil. In addition, under the action of the high temperature generated from electric spark discharge, the copper foil of the PCB is directly eroded through the non-contact machining, thus effectively avoiding the generation of hole mouth burr. The copper foil of PCB is machined by micro-EDM. Modified resin, ceramic packing, and glass fiber cloth are not conductive, so they can only be machined by mechanical drilling with the micro-bit. The specific technical process is shown in Figure 2.

In the machining process, the micro-bit is connected to the negative pole of the pulse power supply, and the copper foil of the PCB is connected with the positive pole. Under the action of the motorized spindle, the rapidly rotating micro-bit gradually approaches the first layer of copper foil. When the distance between the micro-bit and the copper foil reaches the discharge gap, an electric spark discharge is generated between them. Under the action of the electric spark discharge, the copper foil is gradually eroded, thus forming the micro-hole. After the copper foil machining is completed, the micro-bit continues to feed to perform mechanical drilling of the modified resin, ceramic packing, and glass fiber cloth.

## 3. Experimental Materials and Equipment

**Figure 3:**
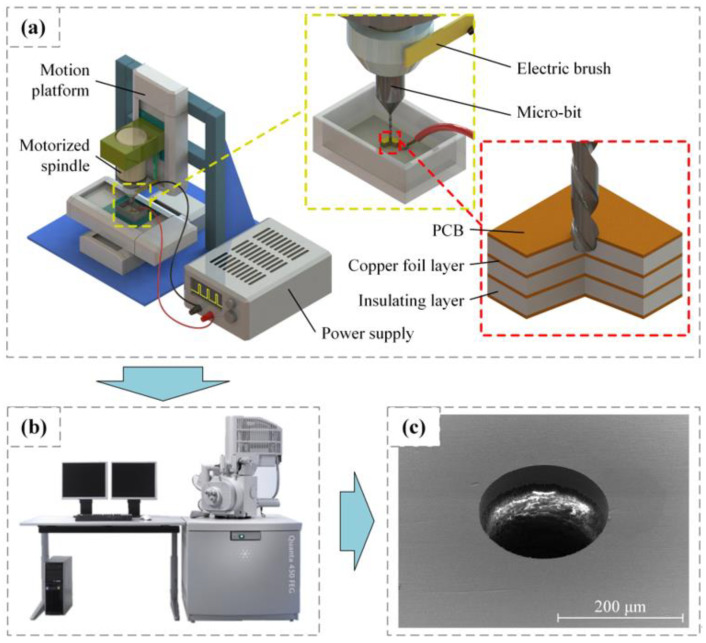
Experimental equipment: (**a**) experimental platform, (**b**) scanning electron microscope, (**c**) micrograph of PCB micro-hole.

The experimental device is shown in Figure 3. It was built using a precision motion platform (M511.DD, PI, Karlsruhe, Germany) with repeated positioning accuracy of 0.1 μm. The speed range of the motorized spindle (BM-320F, NAKANISHI, Kanuma, Japan) was from 1000 to 80,000 rpm. The geometric parameters of micro-bit (1101220N, AOSHI ELECTRONIC, Shenzhen, China) used in this paper are shown in Table 1. A power supply self-made in the laboratory (SZU-01, Shenzhen University, Shenzhen, China) was used as the pulse power supply, which has a voltage regulation range from 0 to 200 V and minimum pulse width of 1 μs. The machining medium was kerosene (EDM-XTRA, Mobil, Irving, TX, USA). After machining, the PCB was placed in absolute ethanol for ultrasonic cleaning. After that, the PCB micro-hole was observed using a scanning electron microscope (Quanta FEG450, FEI, Hillsboro, OR, USA). When observing the PCB micro-holes, the scanning electron microscope could only capture conductive objects (copper foil layer), so the insulating layer appears as a white area in the micrograph (see Figure 3c).

## 4. Results and Discussion

In the micro-EDM of the copper foil, the rotation of the micro-bit was conducive to the renewal of spark oil in the machining area and the removal of electric erosion products [24]. At the same time, the high-speed rotation of the micro-bit affected the formation of the discharge channel, which had an important influence on the machining quality of the PCB micro-hole. Machining voltage, pulse width, and duty cycle will affect the discharge energy, which in turn affects the performance characteristics of micro-EDM machining [25]. Therefore, this paper firstly studied the influence of spindle speed on the machining quality of PCB micro-holes. After that, the influence of machining voltage, pulse width, and duty cycle on the machining quality of PCB micro-holes was studied. The range of the main process parameters used in the experiment is shown in Table 2.

### 4.1. Influence of Spindle Speed on the Machining Quality of PCB Micro-Holes

**Figure 4:**
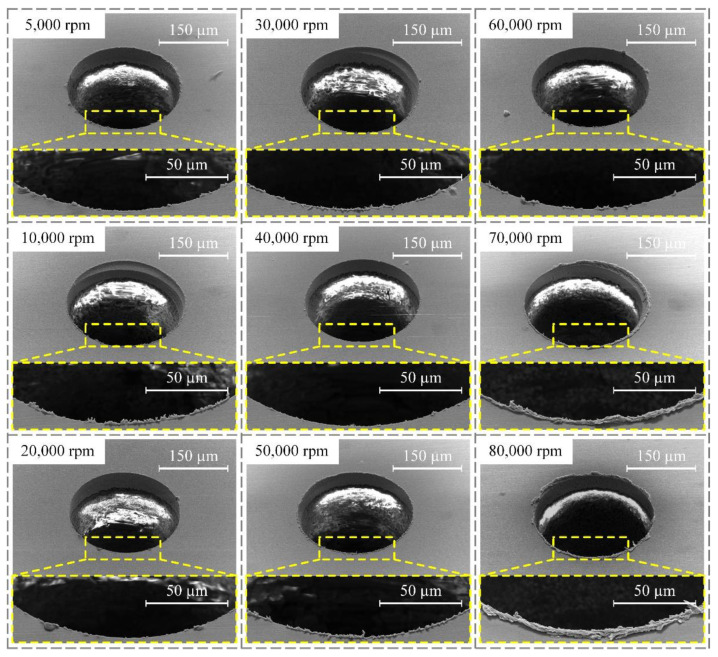
Influence of spindle speed on the quality of micro-hole mouth.

**Figure 5:**
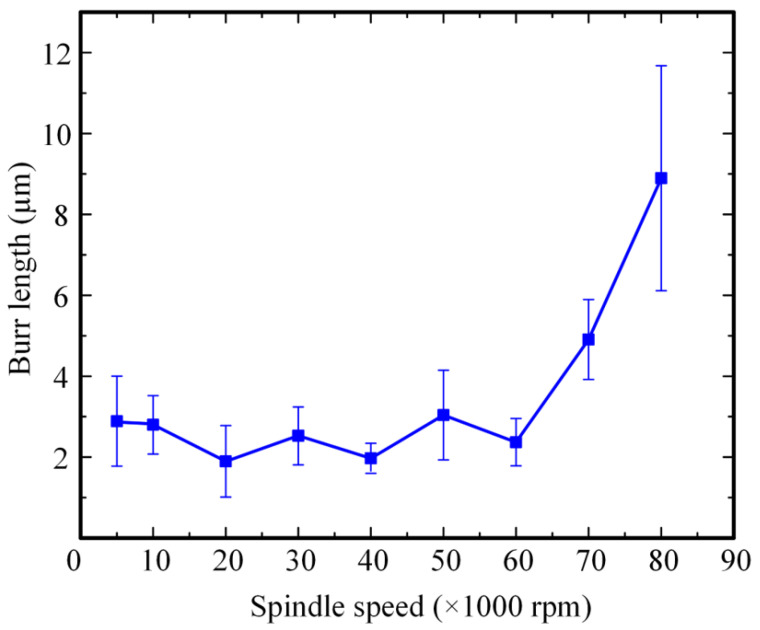
Influence of spindle speed on the average length of the hole mouth burr.

In order to investigate the influence of spindle speed on the machining quality of PCB micro-holes, experiments were carried out under conditions of 24 V machining voltage, 3 μs pulse width, and 6 μs pulse interval. The diameter of the micro-bit was 200 μm, and the feed speed of the micro-bit was 0.2 mm/s. The spindle speed ranged from 5000 to 80,000 rpm, and the experimental results are shown in Figure 4. In order to quantitatively evaluate the hole mouth burr, an area with large burr length within the hole mouth range was selected for measurement. In the area, the lengths of five burrs were measured, and the average value was calculated. The results of the measurement are shown in Figure 5.

As shown in Figure 4, when the spindle speed increased from 5000 to 20,000 rpm, the hole mouth burr became less and less obvious. In the 20,000 to 60,000 rpm range, the hole mouth burr remained very slight. However, when the spindle speed exceeded 70,000 rpm, the hole mouth burr became more and more obvious. When the spindle speed was 5000 rpm, the average length of hole mouth burr was 2.89 μm. As shown in Figure 5, when the spindle speed increased from 5000 to 20,000 rpm, the average length of the hole mouth burr decreased from 2.89 to 1.89 μm. When the spindle speed increased from 30,000 to 50,000 rpm, the maximum and minimum average lengths of the hole mouth burr were 3.04 and 1.97 μm, respectively. When the spindle speed increased from 60,000 to 80,000 rpm, the average length of the hole mouth burr increased from 2.37 to 8.90 μm.

The rotary movement of the micro-bit was conducive to the renewal of spark oil and the removal of electric erosion products in the machining area, thus improving the efficiency and machining quality of the micro-hole. At the same time, the rotation of the micro-bit had an adverse effect on the formation of the discharge channel between the micro-bit and the copper foil. When the rotation speed of the micro-bit was too high, it was difficult to form a stable discharge channel, which easily generated the unstable spark discharge between the micro-bit and the copper foil. Under the influence of this factor, short circuiting easily happened in micro-EDM, which reduced the machining quality of the micro-hole. In addition, when the rotation speed of the micro-bit was too high, the vibration amplitude and frequency of the experimental platform increased, which was not conducive to performing micro-EDM.

Therefore, when the spindle speed was in the range of 5000 to 20,000 rpm, the rotation of the micro-bit could promote the renewal of spark oil in the machining area and the removal of electric erosion products, thus making the average length of the hole mouth burr gradually smaller. When the spindle speed exceeded 30,000 rpm, the rotation of the micro-bit was not conducive to the stable performance of micro-EDM, which gradually increased the average length of the hole mouth burr. When the spindle speed was 20,000 rpm, the rotation of the micro-bit was conducive to the renewal of spark oil in the machining area and did not adversely affect the generation of discharge channels. At this time, the average length of the hole mouth burr was the smallest (1.89 μm). Therefore, the spindle speed was set to 20,000 rpm in this paper.

### 4.2. Influence of Machining Voltage on the Machining Quality of PCB Micro-Hole

**Figure 6:**
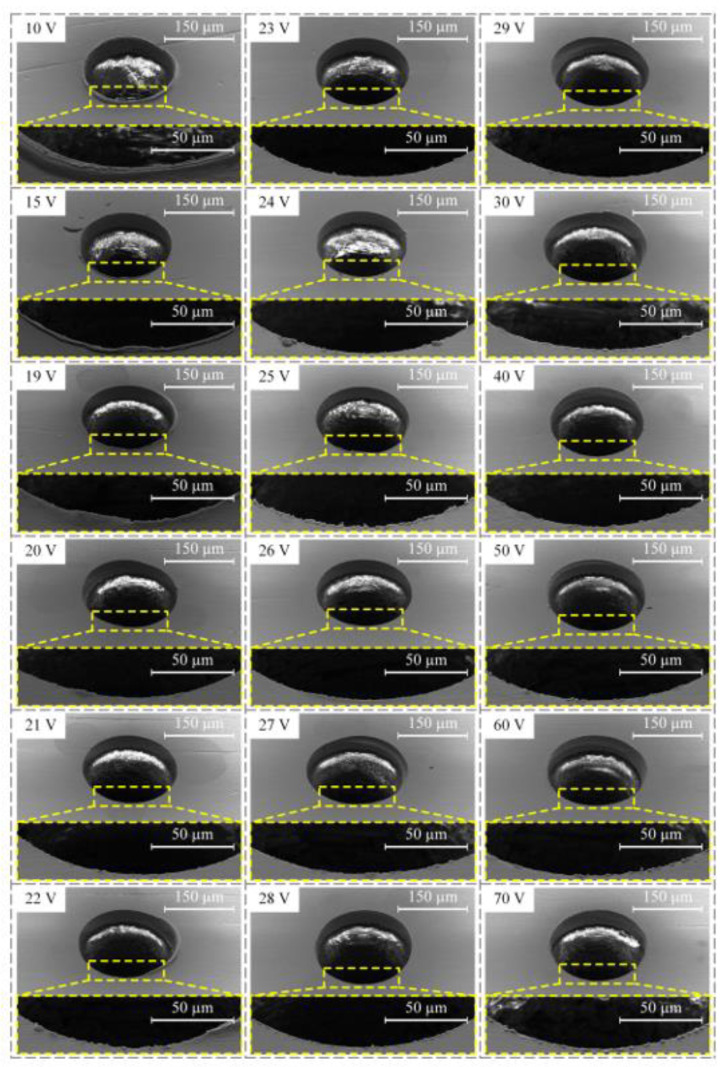
Influence of machining voltage on the machining quality of micro-holes.

**Figure 7:**
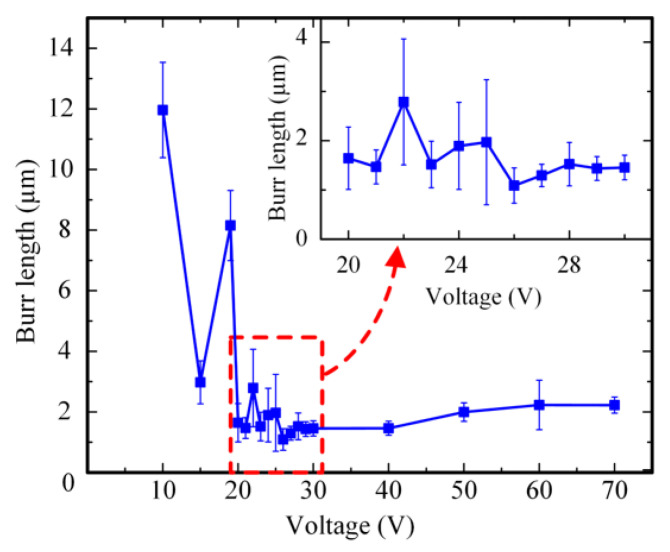
Influence of machining voltage on the average length of the hole mouth burr.

In order to investigate the influence of machining voltage on the machining quality of PCB micro-holes, different machining voltages were used to perform micro-EDM under 20,000 rpm spindle speed, 3 μs pulse width, and 6 μs pulse interval. The diameter of the micro-bit was 200 μm, and the feed speed of the micro-bit was 0.2 mm/s. The machining results are shown in Figure 6. In order to quantitatively evaluate the hole mouth burr, an area with large burr length within the hole mouth range was selected for measurement. In the area, the lengths of five burrs were measured, and the average value was calculated. The results of the measurement are shown in Figure 7.

As shown in Figure 6, when the machining voltages were 10, 15, and 19 V, the hole mouth quality of the PCB micro-hole was poor and featured a legible burr. When the machining voltage was between 20 and 30 V, the hole mouth quality was improved and the hole mouth burr was not obvious. When the machining voltage increased from 30 to 70 V, the hole mouth quality gradually deteriorated, and the hole mouth burr became increasingly obvious. As shown in Figure 7, when the machining voltage was between 10 and 19 V, the maximum and minimum average lengths of the hole mouth burr were 11.96 and 2.98 μm, respectively. When the machining voltage was between 20 and 30 V, the maximum and minimum average lengths of the hole mouth burr were 2.79 and 1.09 μm, respectively. When the machining voltage was between 40 and 70 V, the maximum and minimum average lengths of the hole mouth burrs were 2.23 and 1.46 μm, respectively

When the machining voltage was low, the energy generated by a single pulse discharge was small and thus the efficiency of material removal was low. At this time, the EDM of the micro-hole was unstable and short circuiting could easily occur. When the short circuit occurred, mechanical friction occurred between the micro-bit and the copper foil. Under these conditions, plastic deformation of the copper foil happened, and thus the hole mouth burr was created. Therefore, when the machining voltage was low, the average length of the hole mouth burr was larger. As the machining voltage increased, the single-pulse discharge energy and the material removal efficiency gradually increased, which made the discharge process gradually stabilize. When the machining voltage was 26 V, the hole mouth quality was the most optimal, and the average length of the hole mouth burr was the smallest (1.09 μm). When the machining voltage was further increased, the single pulse discharge energy continued to increase, and the discharge pit generated from micro-EDM became larger. Meanwhile, the molten copper splashed, cooled, and adhered to the hole mouth under the action of the micro-bit rotation. Consequently, the hole mouth burr became more and more obvious. Therefore, when the machining voltage was too high, the hole mouth quality decreased, and the hole mouth burr became obvious. In order to ensure the machining quality of the micro-hole, the machining voltage was set to 26 V in this paper.

### 4.3. Influence of Pulse Width and Duty Cycle on the Machining Quality of PCB Micro-Hole

**Figure 8:**
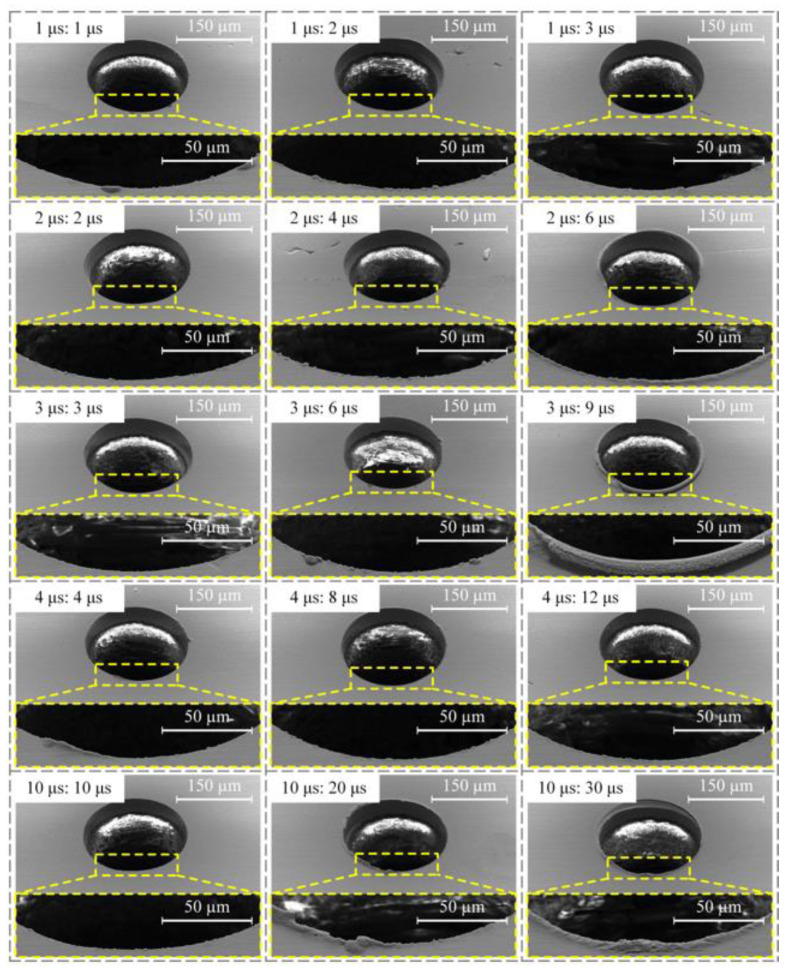
Influence of pulse width and duty cycle on the machining quality of micro-hole.

**Figure 9:**
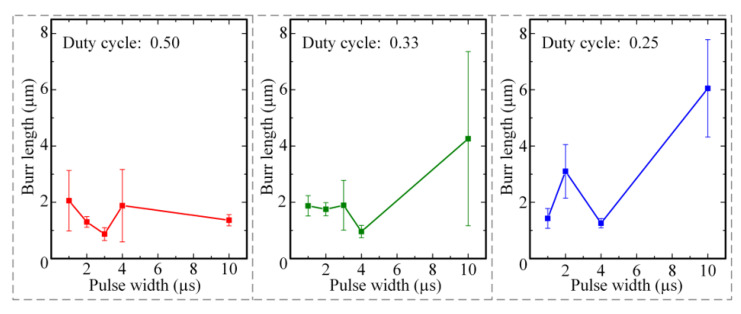
Influence of pulse width and pulse duty cycle on the average length of the hole mouth burrs.

In order to study the influence of pulse width and duty cycle on the machining quality of micro-hole, this paper used different pulse widths and duty cycles to perform micro-EDM of a micro-hole under the effects of 20,000 rpm spindle speed and 26 V machining voltage. The pulse widths were set to 1, 2, 3, 4, and 10 μs. The duty cycle was 0.50, 0.33, and 0.25, respectively. The diameter of the micro-bit was 200 μm, and the feed speed of the micro-bit was 0.2 mm/s. The results of the machining are shown in Figure 8. In Figure 8, the different pulse widths: pulse intervals were marked in the upper left corner of each micrograph. In order to quantitatively evaluate the hole mouth burr, an area with large burr length within the hole mouth range was selected for measurement. In the area, the lengths of five burrs were measured, and the average value was calculated. The results of the measurement are shown in Figure 9.

As shown in Figure 8, when the pulse width was 3 μs and the duty cycle was 0.25, the machining quality of the PCB micro-hole was the worst and the hole mouth burr was the most obvious. When the pulse width was 10 μs and the duty cycles were 0.33 and 0.25, the hole mouth quality of the PCB micro-hole was poor and the hole mouth burr was obvious. As shown in Figure 9, when the duty cycle was 0.50, the maximum and minimum average lengths of the hole mouth burr were 2.06 and 0.87 μm, respectively. When the duty cycle was 0.33, the maximum and minimum average lengths of hole mouth burr were 4.26 and 0.96 μm, respectively. When the duty cycle was 0.25, the maximum and minimum average lengths of the hole mouth burr were 6.05 and 1.26 μm, respectively. From the above experimental results, it can be seen that when the pulse width was 3 μs and the duty cycle was 0.50, the average length of the hole mouth burr was the smallest (0.87 μm). When the pulse width was 4 μs and the duty cycle was 0.33, the average length of hole mouth burr was the second smallest (0.96 μm).

When the pulse width was small, the single pulse discharge energy was small and thus the material removal efficiency was low, which caused the micro-EDM discharge instability. In this case, the short circuit easily happened in micro-EDM, and the hole mouth burr was created. With the increase of pulse width, the single pulse discharge energy increased gradually, and material removal efficiency was improved. At this time, the micro-EDM became stable. However, when the pulse width was further increased, the single pulse discharge energy increased, which made the copper foil material melt violently. Under this condition, the average length of the hole mouth burr increased.

**Figure 10:**
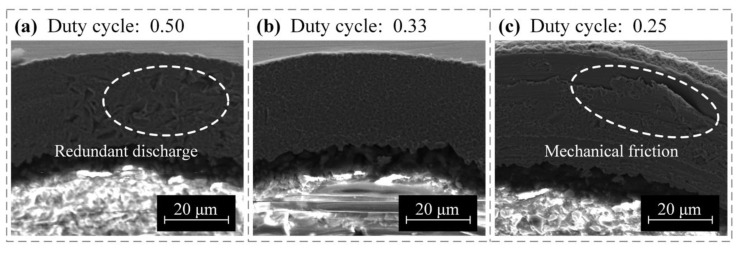
Influence of duty cycle on the machining quality of the interior wall of the micro-hole: (**a**) duty cycle was 0.50, (**b**) duty cycle was 0.33, and (**c**) duty cycle was 0.25.

During the pulse period, electric discharge continuously occurred between the micro-bit and the copper foil, thereby completing the erosion of the copper foil material. During the pulse interval period, there was no discharge between the micro-bit and the copper foil, so the electric erosion products were discharged and the machining medium was deionized. Therefore, when the duty cycle was 0.50, there was not enough time for the electric erosion products to be completely discharged. Under this working condition, the discharge process was unstable, and the machining efficiency was low. In addition, due to the insufficient discharge of electric erosion products, these byproducts can easily accumulate in the machining area, resulting in secondary discharge and eventually reduced machining quality of the interior wall of the micro-hole (see Figure 10a). When the duty cycle was 0.33, the electrical erosion products were fully discharged, so the average lengths of the hole mouth burr were smaller and the machining quality of the interior wall of the micro-hole was improved (see Figure 10b). When the duty cycle was 0.25, the discharge time per unit time decreased and the material removal efficiency was low. Under this working condition, the micro-EDM for producing the micro-hole was unstable and short circuiting could easily occur. As a result, the mechanical friction between the micro-bit and the copper foil occurred, and the machining quality of the micro-hole was decreased (see Figure 10c). Considering the machining efficiency, the average length of the hole mouth burr, and the machining quality of micro-hole, the pulse width and duty cycle were set to 4 μs and 0.33, respectively.

## 5. Elimination of Hole Mouth Burr in Multilayer PCB Micro Hole Based on the Micro-EDM

**Figure 11:**
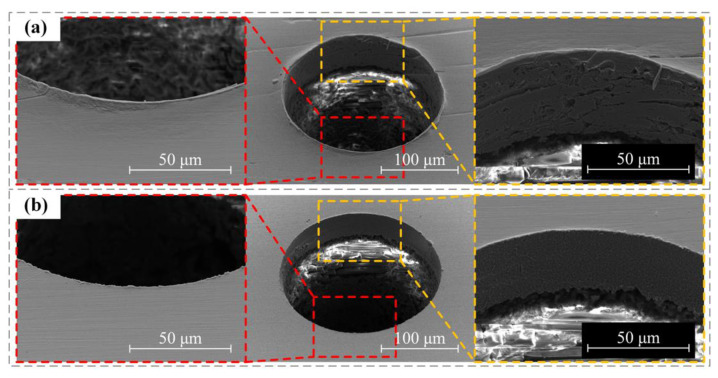
Micrographs of micro-holes machined by the two processes: (**a**) micro-hole machined by mechanical drilling; (**b**) micro-hole machined by micro-EDM.

Previous studies have shown that increasing the spindle speed could improve the hole mouth quality when the mechanical drilling was adopted to machine PCB micro-holes. In the paper, the high speed of the motorized spindle was adopted to mechanically drill the PCB micro-hole, and good machining quality was obtained. Under the effects of 80,000 rpm spindle speed and 2 mm/s feed speed, a micro-bit with a diameter of 200 μm was used to carry out the mechanical drilling of the PCB micro-hole. The results of this machining are shown in Figure 11. As shown in Figure 11a, the overall morphology of the PCB micro-hole obtained by mechanical drilling was poor, with a hole mouth burr and low-quality interior hole wall. As shown in Figure 11b, the PCB micro-hole obtained by micro-EDM showed good overall morphology, with no obvious hole mouth burr and good quality of the interior wall. The above machining results showed that the machining results obtained from high-speed mechanical drilling were worse than those of micro-EDM with low spindle speed. This proved that micro-EDM of PCB micro-hole not only had higher processing quality, but also had lower requirements for spindle speed and energy consumption.

## 6. Conclusions

At present, mechanical drilling based on the micro-bit is the most common technique for the machining of PCB micro-holes. However, in the mechanical drilling of micro-holes, the mouth of the micro-hole is prone to burring due to the micro-bit failing to cleanly cut through the first layer of copper foil on the PCB. A hole mouth burr can potentially cause short circuiting and even lead to ruining the PCB. To solve the above problem, this paper proposed to machine the first layer of copper foil via micro-EDM to eliminate the hole mouth burr. Through detailed study, using micro-EDM to machine PCB micro-holes can effectively eliminate hole mouth burrs and improve the quality of PCB micro-holes. That provides a new possibility for PCB micro-hole machining technology. The conclusions are as follows:

1. Distinct from mechanical drilling technology, micro-EDM is a non-contact machining technique. Under the action of high temperature generated from electric spark discharge, the first layer of copper foil can be directly eroded, thus effectively avoiding the generation of hole mouth burrs.
2. Under the effects of 20,000 rpm spindle speed, 26 V machining voltage, 4 μs pulse width, 8 μs pulse interval, and 0.2 mm/s feed speed, a micro-bit with 200 μm diameter was used to machine micro-holes in PCB. From the machining results, it can be seen that the PCB micro-hole obtained by micro-EDM has good overall morphology, high quality of interior walls, and an average burr length of 0.96 μm.
3. Compared with mechanical drilling, micro-EDM of PCB micro-holes can eliminate the hole mouth burr and improve the machining quality of the hole mouth. However, micro-EDM of PCB micro-holes requires more operation steps, which may increase the cost of manufacturing. When PCB micro-holes require good machining quality, micro-EDM is suggested for machining the PCB micro-hole.

## Figures and Tables

**Table 1:** Geometric parameters of micro-bit used in the paper.

Parameters	Value
Diameter	0.2 mm
Number of cutting edges	2
Length of cutting edge	2.5 mm
Point angle	130°

**Table 2:** Main process parameters used in the paper.

Process Parameters	Range of Process Parameters
Spindle speed	1000–80,000 rpm
Machining voltage	10–80 V
Pulse width	1–10 μs
Duty cycle	0.50; 0.33; 0.25
Feed speed	0.2 mm/s
